# Multimodal insights into diverse pain experiences: PhysioPain dataset

**DOI:** 10.1016/j.dib.2025.111992

**Published:** 2025-08-19

**Authors:** Boran Toktay, İkbal Işık Orhan, Elif Yıldırım, Fatma Patlar Akbulut, Cagatay Catal

**Affiliations:** a Department of Computer Engineering, Istanbul Kültür University, Istanbul, Türkiye; b Department of Computer Engineering, Istanbul Technical University, Istanbul, Türkiye; c Department of Software Engineering, Istanbul Kültür University, Istanbul, Türkiye; d Department of Computer Science and Engineering, College of Engineering, Qatar University, Doha, Qatar

**Keywords:** Multimodal pain assessment, Physiological signal analysis, Pain classification, Biosignals, Pain intensity estimation

## Abstract

PhysioPain dataset comprises several physiological data of different kinds of pain: no pain, headache, menstrual cycle pain and back/neck/waist pain in search of a sophisticated and complete approach to pain representation. The study comprised 99 individuals, of whom 93 participants contributed real-time physiological data. These participants underwent experiment process to gather real-time physiological data including electroencephalogram (EEG), skin temperature, electrodermal activity (EDA), blood volume pulse (BVP), and accelerometer data. Combining objective physiological data with subjective information acquired by the survey using the McGill questionnaire and customized questions produces a complete dataset fit for the tasks related to pain estimate, pain classification, and other approaches to pain observation. This method seeks to offer a fresh viewpoint on pain intensity and catch a more complete knowledge of the intricate character of pain experiences.

Specifications Table

**Table:** 

Subject	Data Science Computer Science Health and medical sciences
Specific subject area	Pain analysis, Pain recognition, Signal Processing, Biosignal classification
Type of data	Raw, Analysed
Data collection	The project took advantage of the Empatica E4 wrist-worn sensor device and NeuroSky's MindWave Mobile EEG device. At 1 Hz, the MindWave detects brainwaves (alpha, beta, gamma, theta) and attention/relaxation levels providing a reasonably affordable and user-friendly configuration. The wrist-worn sensor device documents 4 Hz EDA, HR, 64 Hz BVP, and ACC. Data collecting took thirty minutes: 20 min of physiological signals then a 10-minute survey covering demographics, emotional factors, and the McGill Pain Questionnaire. Separately stored, arranged by pain type, and resampled to produce different datasets were signals and survey data.
Data source location	Institution: Istanbul Kültür University, Department of Computer Engineering City/Town/Region: Istanbul, Bakırköy, Ataköy Country: Turkey Latitude and longitude (and GPS coordinates, if possible) for collected samples/data: 41° 5 7.3284′’ N 29° 2 22.3836 ’ E
Data accessibility	Repository name: Mendeley Data Data identification number: 10.17632/mf2cgph9cy.4 Direct URL to data: https://data.mendeley.com/datasets/mf2cgph9cy/4
Related research article	None

## 1 Value of the Data

• The PhysioPain dataset provides a unique tool for examining the complex interaction between subjective and objective elements of pain by integrating self-reported data from participants with physiological signals (EEG, EDA, BVP, HR, and ACC). This facilitates a more comprehensive understanding of the dynamics of pain.
• This dataset provides researchers the opportunity to develop and verify pain classification models, improve pain management strategies, and develop personalized healthcare interventions by incorporating a variety of pain types (headache, menstrual pain, back/neck/waist pain, and non-pain).
• The dataset combines responses to the McGill Pain Questionnaire with signals from wearable devices, so offering reusable, real-world data for validation of bio-signal processing algorithms and machine learning models in pain assessment.
• Using the data, researchers in disciplines including neuroscience, psychology, biomedical engineering, and artificial intelligence can investigate pain detection, assess signal processing methods, and progress individualized pain management systems.
• By means of advanced AI-driven models, the dataset supports research aiming at identifying pain in populations unable to self-report, including children, elderly, or patients with communication difficulties, so promoting early diagnosis and treatment.

## 2 Background

Because pain is subjective and combines sensory, cognitive, and emotional elements, assessment of it is difficult. Conventional pain assessment paradigms are sometimes beset with inherent constraints including the inclination for observer subjectivity and variance and self-reporting biases resulting from individual perceptual variances. Several studies had been conducted to represent pain in a more objective way by using bio-signals [[1], [2], [3], [4]], but new problems raised including inter-subject variability and time-dynamic differences in pain behaviours, limited cross-subject generalization, insufficient adaptability to variability, and a limited understanding of the precise temporal dynamics of pain signals. However, existing datasets are not very useful for thorough pain analysis since they usually miss the interaction between subjective experiences and physiological reactions.

The PhysioPain dataset was formed using a multimodal approach combining self-reported pain characteristics with physiological data including EEG, EDA, BVP, HR, and ACC to help to address these shortcomings. Ensuring broad applicability across several pain conditions, the dataset comprises data from participants experiencing various pain types—headache, menstrual pain, back/neck/waist pain, or no pain. Furthermore giving a subjective view of pain experiences are demographic studies and the McGill Pain Questionnaire. This structure ensures a thorough collection ready for the analysis of the complex and changing character of pain. By combining subjective and objective data, the dataset seeks to enable improvements in pain estimate, classification, and tailored treatment plans.

## 3 Data Description

**Fig. 1:**
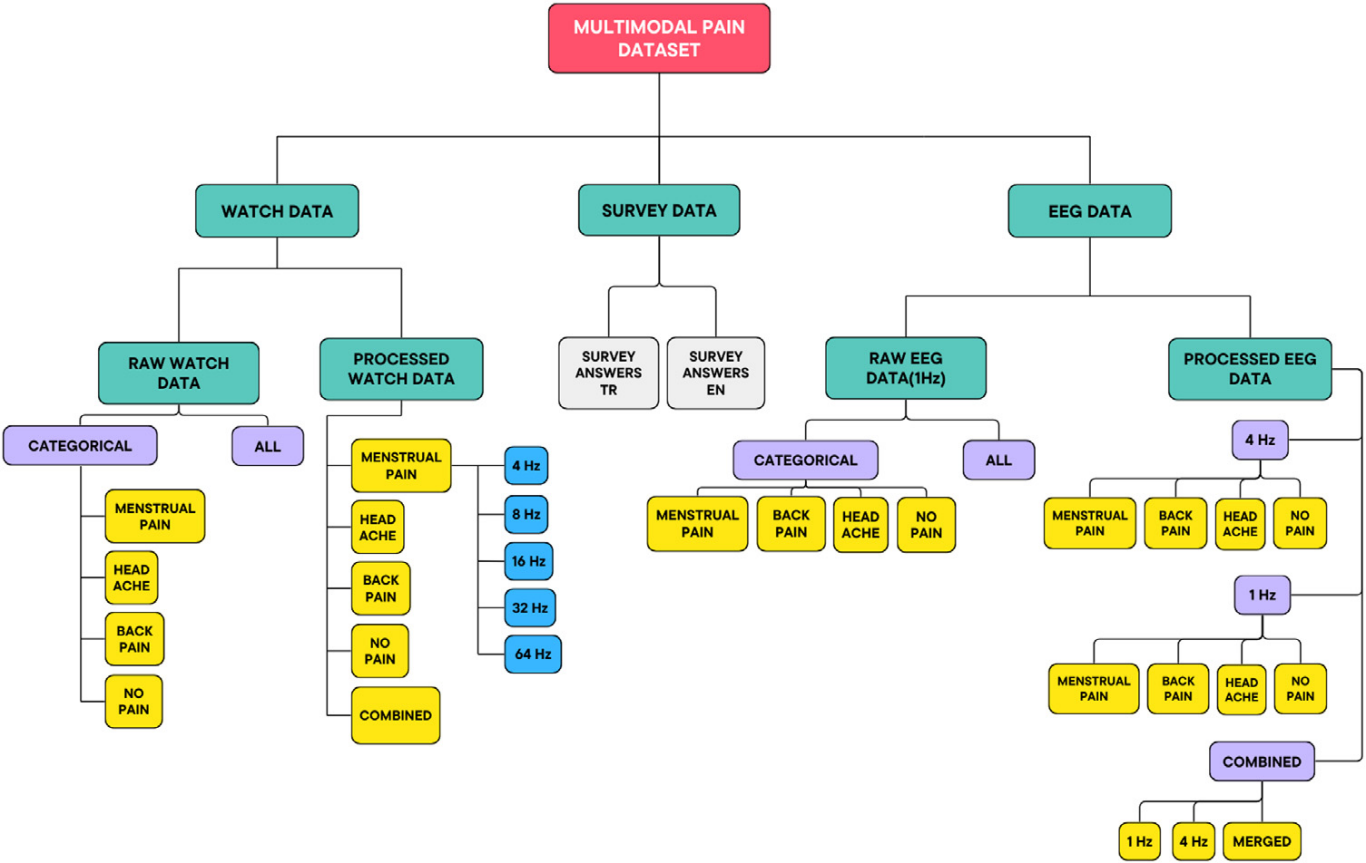
General structure and folder clustering of multimodal PhysioPain dataset.

1200 samples of signal data (for 1 Hz) collected for 20 min from EEG and wristworn sensor devices and survey is evaluated by the participants to expand our understanding of pain for 3 different types of pain including headache, menstrual pain and back/neck/waist pain. The survey consists of 118 questions divided into 3 parts. The first part includes demographic questions such as age, gender, and income. In order to learn about the participant's daily activities and habits, questions like how much water the participant drinks in general and before the test, whether he/she smokes or drinks alcohol, how much sleep he/she gets are asked. Then, the survey continues with the McGill pain questionnaire in the second part. The last part of the survey includes pain type specific questions. Objective physiological data of the participants such as various bio signals including EEG, EDA, BVP (Blood Volume Pulse), body temperature and accelerometer are included in the dataset in both raw and processed versions. Fig. 1 shows the general structure and the folder clustering of the dataset.

Fig. 1 illustrates the folder structure of the dataset. The detailed structure of folders, subfolders and files of the dataset are given in the sections below:

a. Survey Data Subfolder: This subfolder contains two different files which contain demographic information, daily habits and answers to the McGill Pain Questionnaire of the participants.
  • Survey_answers_TR.xlsx: Answers of the participants in Turkish.
  • Survey_answers_EN.xlsx: Answers of the participants in English.
b. Watch Data: Includes all watch data.

b.1. Raw Watch Data Subfolder: This subfolder of the dataset shows the raw data collected by wrist-worn sensor device and it includes 4 Hz EDA signals, 64 Hz BVP signals, 32 Hz accelerometer data (X, Y) signals, 4 Hz temperature signals, HR, IBI, tags and info files of the participants.
  b.1.1. Categorical Subfolder: This subfolder contains categorized information of the participants according to their pain types.
    • menstrual_pain: Raw wristband data of the participants who suffer from menstrual pain according to their IDs (ex: S001.zip, S002.zip, …).
    • no_pain: Raw wristband data of the participants who do not experience any pain according to their IDs.
    • back_pain: Raw wristband data of the participants who suffer from back pain according to their IDs.
    • headache: Raw wristband data of the participants who suffer from headache according to their IDs.
  b.1.2. All Subfolder: Contains all participants’ raw wristband data.

b.2. Processed Watch Data Subfolder: In wrist-worn sensor device, each data collection exhibits a distinct sampling frequency. By using upsampling and downsampling methods, the data was resampled for sampling frequency consistency. Therefore, BVP, EDA, ACC(x,y,z), temperature data are stored in one dataset labelled with pain scale, pain type, and person ID information. Multiple datasets are created to represent data in 5 different frequency levels (4 Hz, 8 Hz, 16 Hz, 32 Hz, 64 Hz) for each pain type.
  b.2.1. menstrual_pain Subfolder: Stores the processed watch data of the participants who suffer from menstrual cycle pain by their unique IDs, the information represented in different frequency levels, therefore, the data stored in subfolders named signal_4, signal_8, signal_16, signal_32, signal_64.
  b.2.2. no_pain Subfolder: Stores the processed watch data of the participants who do not experience any pain by their unique IDs, the information represented in different frequency levels, therefore, the data stored in subfolders named signal_4, signal_8, signal_16, signal_32, signal_64.
  b.2.3. back_pain Subfolder: Stores the processed watch data of the participants who suffer from back pain by their unique IDs, the information represented in different frequency levels, therefore, the data stored in subfolders named signal_4, signal_8, signal_16, signal_32, signal_64.
  b.2.4. Headache Subfolder: Stores the processed watch data of the participants who suffer from headache by their unique IDs, the information represented in different frequency levels, therefore, the data stored in subfolders named signal_4, signal_8, signal_16, signal_32, signal_64.
  b.2.5. Combined Subfolder: Combination of each participants’ processed watch data stored in .csv files for different types of pain and different frequencies.
    • With No Pain Subfolder: This subfolder includes datasets that combines the “no pain” processed watch data with different pain categories for distinct frequencies.
    • No Pain Subfolder: Combination of all participants’ processed watch data who do not experience any pain represented in different frequencies.
    • Menstrual Pain Subfolder: Combination of all participants’ processed watch data who experience menstrual pain represented in different frequencies.
    • Headache Pain Subfolder: Combination of all participants’ processed watch data who experience headache represented in different frequencies.
    • Back Pain Subfolder: Combination of all participants’ processed watch data who experience back pain represented in different frequencies.
    • Combined All Data Subfolder: Combination of all participants’ processed watch data in different frequencies.
c. EEG Data: Includes all EEG data.
  c.1. Raw EEG Data (1 Hz) Subfolder: This subfolder contains 1 Hz EEG data including alpha, beta, theta, gamma brain waves of the participants.
    c.1.1. Categorical Subfolder: This subfolder contains categorized information of the participants according to their pain types.
      • menstrual_pain: Raw EEG data of the participants who suffer from menstrual pain according to their IDs (ex: S001.csv, S002.csv, …).
      • no_pain: Raw EEG data of the participants who do not experience any pain according to their IDs.
      • back_pain: Raw EEG data of the participants who suffer from back pain according to their IDs.
      • headache: Raw EEG data of the participants who suffer from headache according to their IDs.
    c.1.2. All Subfolder: Contains all participants’ raw EEG data.
  c.2. Processed EEG Data Subfolder: This subfolder contains processed EEG data of the participants with different frequency levels. In processed EEG data, bio-signals are labelled with pain types and pain intensity.
    c.2.1. 1 Hz Subfolder: It contains headache, back pain, menstrual pain and no pain subfolders, within each folder, 1 Hz processed EEG data included for each participant.
    c.2.2. 4 Hz Subfolder: It contains headache, back pain, menstrual pain and no pain subfolders, within each folder, 4 Hz processed EEG data included for each participant.
    c.2.3. Combined: The combined data of processed EEG includes all participants’ information together, represented in both 1 Hz and 4 Hz signals and different categories.

A wearable smartwatch provided the physiological data shown in Table 1. A person in pain is experiencing a range of bodily responses included in this dataset: BVP, EDA, and TEMP along with pain scale and pain type data. Often used in psychophysiological studies, these measures provide information on the physical activity levels and responses of the autonomic nervous system in the body. Knowing the temporal dynamics and interrelationships of these signals helps one to grasp the physiological reactions in the framework of the research.

**Table 1:** Detailed information of the columns in the preprocessed wrist-worn sensor device dataset.

Data	Data Type	Data Description
BVP	float	It is the signal that measures the heart rate. Heart rate changes that occur when the level of stress or tension is high can be detected with this signal.
EDA	float	It is the signal data used to measure the electrical activity of the skin. Resistance on the skin surface can vary depending on perspiration, stress, mood, physical activity, and other factors.
TEMP	float	Signal data used to measure the surface temperature of the skin. This signal data is used to monitor how stressed the individual is, exercise and health status.
pain_scale	integer	the level of pain experienced by the participant
pain_type	object	the type of pain the participant has

The EEG data acquired in this study is shown in Table 2. This component of the dataset consists of several frequency bands of the brain's electrical activity, including delta, theta, alpha1, alpha2, beta1, beta2, gamma1, and gamma2 signals. Different cognitive states and neural processes are linked to these separate frequency bands.

**Table 2:** Detailed information of the columns in the preprocessed EEG dataset.

Data	Data Type	Data Description
Delta	integer	High frequency brain waves during rest and deep sleep
Theta	integer	It is characterized as a brain wave that produces a high frequency during the transition to sleep.
Alpha1	integer	It is a brain wave, a subdivision of alpha waves, which manifests itself in moments of cognitive relaxation, yawning and a decrease in stress levels.
Alpha2	integer	It is a brain wave associated with cognitive concentration and attention and is another subdivision of alpha.
Beta1	float	It is a derivative of beta waves, which are characterized by increased cognitive effort.
Beta2	float	It is another derivative of beta waves. It is a brain wave that enables cognitive alertness and attention, as well as inferring information about memory
Gamma1	float	It is a derivative of gamma brain waves, which are associated with cognitive activities such as attention, memory, problem solving and learning new information.
Gamma2	float	It is a wave type that generates higher frequency than gamma1 waves during cognitive performance such as attention, memory, problem solving and learning new information.
pain_version	object	the type of pain the participant has
pain_intensity	float	the level of pain experienced by the participant
person_id	float	person id of the participant

## 4 Experimental Design, Materials and Methods

### 4.1 Research design

While creating PhysioPain, it is aimed to bring a more objective and generalized perspective of pain by collecting multimodal data while they experience pain. The participants come from diverse professional and educational backgrounds, with the majority aged between 18 and 26. Each participant contributed to the study in a single 30–35-minute session, conducted at different times of the day. Empatica E4 and NeuroSky Mindwave EEG devices were operated simultaneously, and the measurement lasted in 20 min to capture signals at different levels of pain while the participant answered the survey which takes 10–15 min. The participants were requested to remove metals such as earrings as they would affect the reliable measurement of the signals and participants were asked to remain as still as possible during the measurement. During the survey, participants answered demographic questions first to assess biological factors such as age and gender, as well as questions to assess how daily life activities and habits affect the sensation or character of pain, such as smoking and alcohol usage, drinking water and eating habits. Following the personal questions in the survey, a short version of McGill Pain Questionnaire is used to examine participants' perceptions of each pain group: headache, menstrual pain, back/neck/waist pain [12,13]. The questionnaire consisted of 3 main parts intended to specify the subjective pain experience of the participants.

In the first part, the participants stated their pain type, and they were asked the following questions using a 5-point Likert scale in the range of 1–5 to analyse how pain feels by the participants:

1. Rate the severity of your pain (Likert scale) [How severe is your pain now?]
2. Rate the severity of your pain (Likert scale) [How bothersome is your pain right now?]
3. Rate the severity of your pain (Likert scale) [How much does your pain currently affect your daily activities?]
4. Rate the severity of your pain (Likert scale) [How unbearable is your pain right now?]
5. Rate the severity of your pain (Likert scale) [How widespread is your pain now?]
6. Moving on to the second part, participants were provided with 20 distinct groups of descriptive words and instructed to choose the words that most accurately describe their pain within each group.
7. In the last part of the questionnaire, participants were asked how does their pain change over time.

**Fig. 2:**
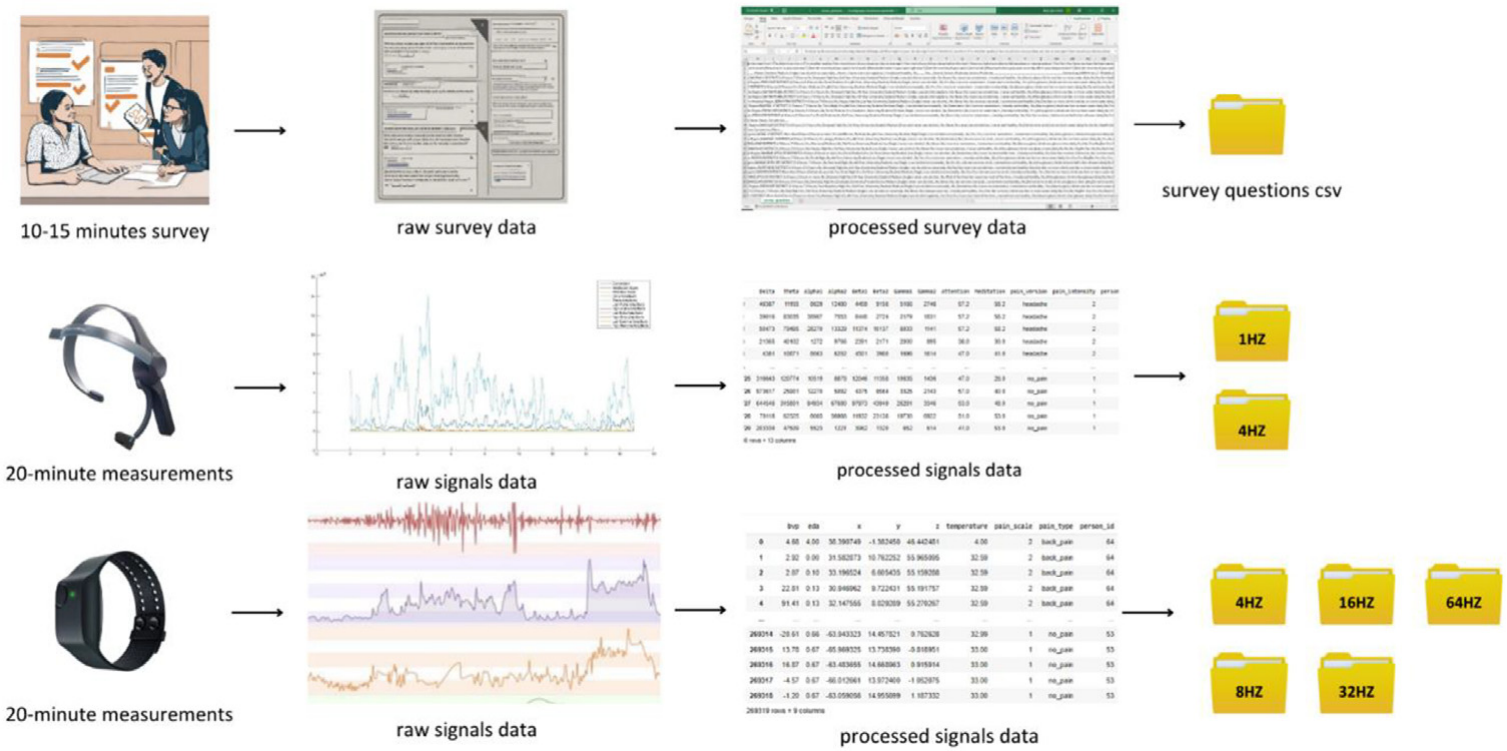
Research design of acquiring data.

The signal data collected for each different pain type were stored in raw form in different files for each participant. Then, the new datasets of processed signals generated by the upsampling and downsampling methods are stored in separate files for each Hz level. The survey data is stored in a single .csv file as it is seen in the Fig. 2.

### 4.2 Measurements of biosignals

The measurement devices utilized in this study include headset sensor device and wrist-worn sensor device equipment. The headset sensor device is a commercial EEG device specifically designed for measuring brain waves. It effectively detects signals such as alpha, beta, gamma, and theta, as well as attention and relaxation levels. This EEG data acquisition is cost-effective and user-friendly, involving attachment to the earlobes and forehead, serving as a voltmeter at the microvolt level with the assistance of electrodes. In conjunction with both devices is employed to measure additional physiological parameters crucial for the dataset. The wrist-worn sensor device captures ACC, BVP, HR, and EDA. By combining EEG and other bio-signal data, it is aimed to develop a comprehensive dataset to be used in studies related to pain analysis.

### 4.3 Data preprocessing

In the data preprocessing stages separate procedures were applied to EEG data and psychological signals. These procedures work synergistically to refine biosignal data for subsequent analysis. The EEG-focused part meticulously establishes a directory structure for organized data storage, addresses missing values, and employs a Gaussian filter to enhance data reliability. Simultaneously, the procedure designed for psychological signals systematically resamples data at various frequencies, applies a Gaussian filter for artifact removal, and organizes the resulting signals into separate directories. Furthermore, lower-frequency physiological signals (1–4 Hz) were resampled to a uniform 4 Hz using interpolation techniques to ensure consistency across the dataset. These preprocessing steps ensured data integrity and facilitated accurate time series classification for both EEG and psychological signals in biosignal research.

Distinct techniques for biosignals defined the data preprocessing pipeline. To guarantee consistency, interpolation was used to resample lower-frequency physiological signals (1–4 Hz) to a uniform 4 Hz. All biosignals such as temperature (4 Hz), accelerometer (32 Hz), blood volume pulse (32 Hz), and electrodermal activity (4 Hz) at different frequencies was resampled. Upsampling and downsampling methods were used to generate several datasets, each standardized to a particular frequency (4, 8, 16, 32, 64 Hz), hence unifying this data. By means of a Gaussian filter [14], artifact removal further refined the resampled signals, therefore enhancing the representation of physiological responses. Then, we organized them into distinct folders by frequency the preprocessed signals formed thorough dataset prepared for time series analysis. Sensor data and their related class labels were combined to produce unified datasets like ``all_head_data_eeg.csv'' for all data.

### 4.4 Study statistics

To ensure data integrity, we applied strict quality-control criteria to all recordings. From 99 participants, 6 were excluded from all subsequent analyses because their physiological recordings were incomplete or did not meet our pre-specified quality thresholds (e.g., > 20 % data loss in any channel, excessive motion artifact in the EDA or EEG, or failure to complete the full McGill questionnaire). Thus, the core analytical cohort consists of the remaining 93 participants whose multimodal data passed quality control and whose survey responses were fully recorded. This study centred on participant, with 53 female and 40 male, at the age of 18 to 26 years representing the predominant age range among the study population in the survey. Based on the answers, it is observed that daily life habits, stress levels, and emotional states had an impact on pain levels by using various statistical tests and regression models.

**Fig. 3:**
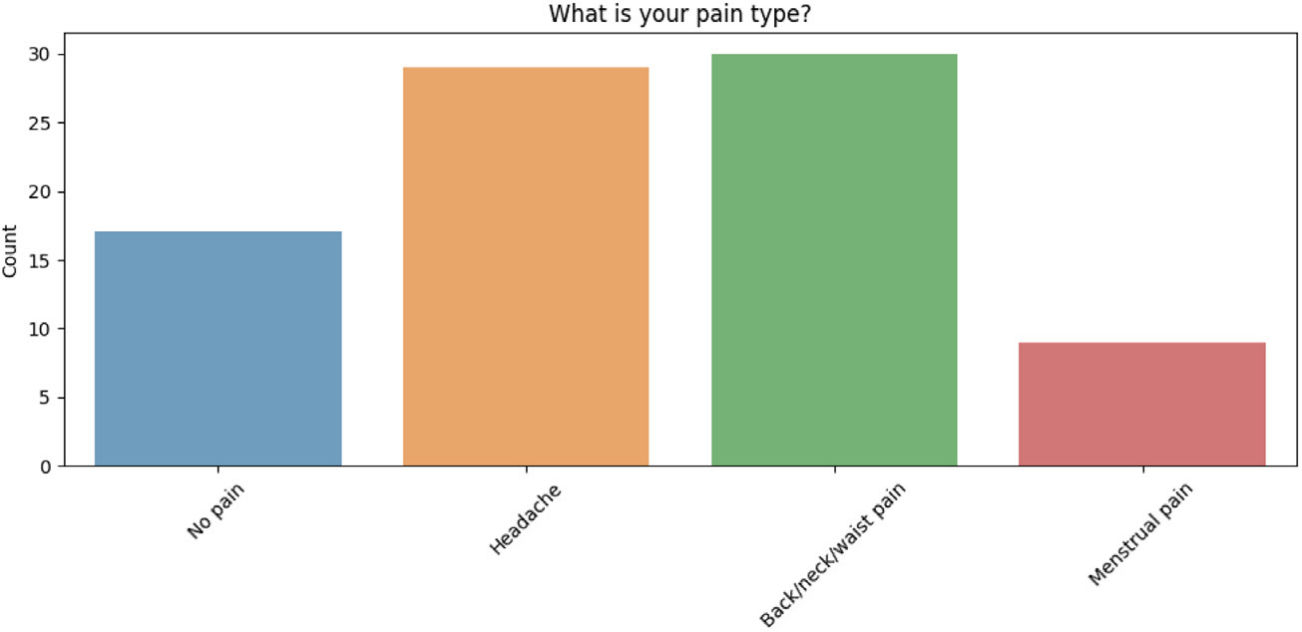
Distribution of the pain types.

**Fig. 4:**
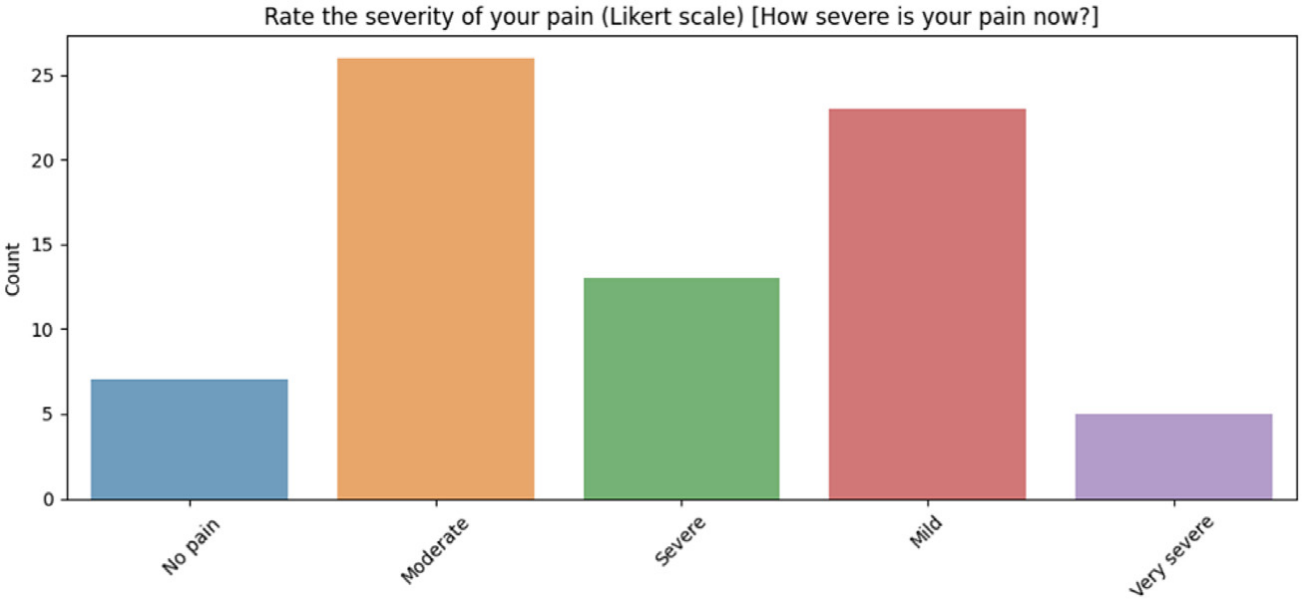
Severity of the pain distribution.

Among the 93 participants, the reported distribution of pain is as follows: 29 individuals stated that they have headache, with pain intensity levels classified as 6 severe, 12 moderate, 10 mild, and 1 no pain as it is seen in the Fig. 3. 11 participants stated that they have menstrual pain, with intensity levels distributed as 2 severe, 3 moderate, 3 mild, and 1 no pain. 30 participants stated that they have pain in the back, neck, or waist, with pain intensity levels categorized as 6 severe, 12 moderate, 12 mild, and 2 no pain. The remaining 19 participants stated that they did not experience any pain during the data collection. Pain severities of all participants are shown in the Fig. 4.

Multiple factors must be considered, such as understanding the neurophysiological mechanisms behind pain as well as the emotional and cognitive variables that affect how pain is felt and expressed [[5], [6], [7], [8], [9]]. It has been established that emotions—especially negative ones—play a significant role in how one perceives and reacts to pain [5,10,11]. Previous studies have shown that those in a state of anxiety or depression experienced a reduction in pain tolerance following induction of negative emotions. The degrees of pain severity were most pronounced in the case of depression [10].

It is observed that sleep patterns, water drinking habits, smoking habits, emotional states and stress levels have an impact on the level of pain. 64 % of the participants stated that they think they do not drink enough water, while 36 % believe they drink enough and 58 % of the participants stated that they generally sleep an average of 6–8 h a day, and 30 % stated that they sleep 4–6 h. The relationship between pain and stress has also examined. Of the participants, 51 % said they were under high stress, 42 % said they were moderate, and 7 % said they were having low levels of stress.

## Limitations

Expanding the dataset with more participants for each pain type and level is of great importance in the development of the dataset. Also, it is essential to acknowledge potential sources of error and anomalies. Human error, particularly in the context of movement interference during data collection, posed a challenge. Additionally, the EEG machine's technical limitations introduced some complexities. Despite these challenges, the overall reliability and utility of the collected data are still robust.

## Ethics Statement

For this study, data were collected with the formal approval of the Ethics Committee of Istanbul Kultur University (dated 05.05.2023 and numbered 2023.66).

## CRediT Author Statement

**Boran Toktay, İkbal Işık Orhan:** Data Collection, Modelling, Data curation, Writing - Original Draft; **Elif Yıldırım:** Validation, Data curation, Writing - Review & Editing; **Fatma Patlar Akbulut:** Conceptualization, Supervision, Methodology, Validation, Writing - Review & Editing. **Cagatay Catal:** Investigation, Writing – review & editing.

## Data Availability

Mendeley DataPhysioPain (Original data).
